# The Impact of Probiotics, Prebiotics, and Functional Foods on Human Health

**DOI:** 10.3390/nu17091529

**Published:** 2025-04-30

**Authors:** Nelson Pérez Guerra

**Affiliations:** Department of Analytical and Food Chemistry, Faculty of Sciences, University of Vigo, 32004 Ourense, Spain; nelsonpg@uvigo.gal

The impact of probiotics, prebiotics, and functional foods on human health continues to be a dynamic and expanding area of research. Numerous studies have explored their potential to modulate gut microbiota, enhance immune function, and address various chronic conditions, highlighting the significant implications of nutritional science in health maintenance and disease prevention. Reflecting this growing interest, this Special Issue presents 15 papers contributed by researchers from diverse fields (including nutrition, biotechnology, biomedicine, clinical pharmacology, metabolic diseases, food science, microbiology, medicine, pediatrics, and neonatology) examining the potential applications of probiotics, prebiotics, and functional foods in promoting human health.

Furthermore, the articles featured in this Special Issue provide compelling evidence that combinations of prebiotics and probiotics, as well as metabolites produced by probiotic strains, can play a valuable role in the management and treatment of various human diseases. For example, the combination of inulin with resistant dextrin (another prebiotic compound) significantly reduced gas production, making it a more tolerable option, particularly for older adults, who are more susceptible to gut microbiota imbalances. Notably, this blend not only enhanced microbial diversity, but also tended to lower the pH in the fermentation medium, indicating a more favorable gut environment (contribution 1).

Thua-Nao, a traditional fermented soybean product from Thailand, demonstrated a capacity to suppress preneoplastic lesions in the liver and colon of rats exposed to carcinogens. The protective effects were associated with both modulation of the gut microbiota and the presence of bioactive compounds such as isoflavones, including genistein and daidzein. These findings suggest the potential of Thua-Nao as a dietary intervention for cancer prevention (contribution 2).

Probiotic supplementation also continues to show promise. A randomized, double-blind trial on Lactobacillus paragasseri OLL2716 revealed that daily consumption of yogurt containing this probiotic significantly alleviated epigastric pain and improved gastrointestinal symptoms. These results support the therapeutic role of probiotics in managing common digestive complaints, such as postprandial fullness and early satiety (contribution 3).

Fruit by-products, such as fiber-rich fractions from persimmon peels and pomace, are also emerging as valuable functional food sources. Rich in phenolic compounds, these fractions promoted the growth of beneficial bacteria like Faecalibacterium prausnitzii, and exhibited anti-inflammatory properties. This study highlights both the potential for food waste valorization, and the role of dietary fibers in supporting both gut health and systemic inflammation reduction (contribution 4).

A combination of Bifidobacterium animalis subsp. lactis GCL2505 with inulin significantly increased resting energy expenditure in overweight individuals. This suggests that modifying the gut microbiota through targeted probiotic and prebiotic intake could enhance metabolic efficiency and support weight management, marking a notable development in the understanding of gut microbiota’s influence on human metabolism (contribution 5).

Sleep deprivation is known to negatively impact gut health, yet pre-colonization with Faecalibacterium prausnitzii was found to protect against intestinal barrier dysfunction in sleep-deprived mice. The treatment reduced inflammation, increased butyrate production, and restored gut microbiota balance. This provides promising evidence for the use of probiotics to mitigate the gastrointestinal effects of poor sleep (contribution 6).

Another compelling study examined the influence of Lacticaseibacillus paracasei strain Shirota on mental performance. Office workers who consumed this probiotic via fermented milk experienced improvements in attention and stress-related physiological markers, such as heart rate variability and theta power on electroencephalogram. These findings reinforce the emerging role of the gut–brain axis in cognitive health and workplace performance (contribution 7).

Further evidence from studies involving GCL2505 and inulin points to their efficacy in reducing visceral and total fat area, as well as in increasing bifidobacteria counts in overweight participants. This supports the hypothesis that gut microbiota modulation can influence fat metabolism and body composition (contribution 8). Additionally, this same combination improved cognitive function, particularly in attention and executive function domains, likely through gut microbiota-mediated reductions in inflammation, offering a novel approach to combat cognitive decline (contribution 9).

Brassica juncea, a glucosinolate-rich plant, has shown potential in addressing metabolic conditions such as obesity and liver disease. Treatments with whole-plant extracts or isolated glucosinolates ameliorated hepatic steatosis and liver injury in high-fat-diet-fed rats. These results suggest that bioactive plant compounds may serve as natural therapeutic agents for non-alcoholic steatohepatitis and related metabolic disorders (contribution 10).

Data from the U.S. National Health and Nutrition Examination Survey (NHANES, 2011–2014) revealed that nonfood probiotics and prebiotics may protect against cognitive decline, particularly in older men. Supplement users exhibited significantly better cognitive scores, suggesting that even non-dietary supplementation may contribute meaningfully to brain health in aging populations (contribution 11).

A preclinical study using a chronic kidney disease (CKD) model demonstrated that resistant maltodextrin and chitosan oligosaccharide supplementation supported gut barrier integrity and promoted beneficial bacteria such as Lactobacillus, Bifidobacteria, Akkermansia, and Roseburia in rats with chronic kidney disease. This intervention may offer a novel strategy to mitigate gut-derived inflammation and complications in CKD (contribution 12).

Comprehensive reviews further emphasize the diversity and specificity of prebiotic and probiotic actions. For instance, inulin and fructo-oligosaccharides (FOS) were shown to prevent microbial dysbiosis, strengthen intestinal barrier function, and modulate immune responses, highlighting the importance of prebiotic variety in maintaining health (contribution 13). In pediatric populations, bioactive compounds derived from probiotics (such as short-chain fatty acids, bacteriocins, exopolysaccharides, vitamins, and gamma-aminobutyric acid) have demonstrated positive effects on immune function, neurodevelopment, and protection against gastrointestinal disorders. These findings reinforce the value of integrating probiotics and postbiotics into child health strategies (contribution 14) [1]. Finally, early-life probiotic intake has shown promise in modulating immune tolerance to food antigens, suggesting a role in both the prevention and treatment of food allergies in infants and children. While mechanisms require further investigation, these studies point to the potential of probiotics in managing allergic diseases (contribution 15) [2]. 

The ingestion of probiotics, prebiotics, or synbiotics in older adults has shown promising results, particularly in improving gut microbiota composition and promoting healthy aging (contributions 1, 3, 5, 9) [3,4]. However, there is conflicting evidence regarding their effects on humoral immunity markers, immune cell population levels and activity, incidence and duration of infectious diseases [3], as well as cognition (contribution 11), physical function, frailty, mood, length of hospitalization, and mortality [5]. Therefore, more rigorous and high-quality research is needed to clarify their broader role in healthy aging and to identify the most effective probiotic combinations [3,4,5].

In summary, this collection of research offers diverse insights into how prebiotics, probiotics, and functional foods may positively influence health outcomes across a wide range of populations and conditions. From metabolic and gastrointestinal health to cognitive function and cancer prevention, the evidence underscores the crucial role of gut microbiota modulation in contemporary nutritional and medical science.

However, as some studies have been conducted using animal models (contributions 2, 6, 10, 12), extrapolating these findings to human health should be approached with great caution, as the results have not yet been validated in humans.

Twenty-eight manuscripts were submitted for consideration for the Special Issue, and all of them were subject to a rigorous review process. In total, fifteen papers were finally accepted for publication and inclusion in this Special Issue (twelve articles and three reviews). The contributions are listed below:

1. Yoshida, K.; Kokubo, E.; Morita, S.; Sonoki, H.; Miyaji, K. Combination of Inulin and Resistant Dextrin Has Superior Prebiotic Effects and Reduces Gas Production During In Vitro Fermentation of Fecal Samples from Older People. *Nutrients* **2024**, *16*, 4262.
2. Taya, S.; Dissook, S.; Ruangsuriya, J.; Yodkeeree, S.; Boonyapranai, K.; Chewonarin, T.; Wongpoomchai, R. Thai Fermented Soybean (Thua-Nao) Prevents Early Stages of Colorectal Carcinogenesis Induced by Diethylnitrosamine and 1,2-Dimethylhydrazine Through Modulations of Cell Proliferation and Gut Microbiota in Rats. *Nutrients* **2024**, *16*, 3506
3. Yamada, N.; Kobayashi, K.; Nagira, A.; Toshimitsu, T.; Sato, A.; Kano, H.; Hojo, K. The Beneficial Effects of Regular Intake of *Lactobacillus paragasseri* OLL2716 on Gastric Discomfort in Healthy Adults: A Randomized, Double-Blind, Placebo-Controlled Study. *Nutrients* **2024**, *16*, 3188.
4. López-Bermudo, L.; Moreno-Chamba, B.; Salazar-Bermeo, J.; Hayward, N.; Morris, A.; Duncan, G.; Russell, W.; Cárdenas, A.; Ortega, Á.; Escudero-López, B.; Berná, G.; Martí Bruña, N.; Duncan, S.; Neacsu, M.; Martin, F. Persimmon Fiber-Rich Ingredients Promote Anti-Inflammatory Responses and the Growth of Beneficial Anti-Inflammatory Firmicutes Species from the Human Colon. *Nutrients* **2024**, *16*, 2518.
5. Baba, Y.; Azuma, N.; Saito, Y.; Takahashi, K.; Matsui, R.; Takara, T. Effect of Intake of Bifidobacteria and Dietary Fiber on Resting Energy Expenditure: A Randomized, Placebo-Controlled, Double-Blind, Parallel-Group Comparison Study. *Nutrients* **2024**, *16*, 2345.
6. Wang, X.; Li, Y.; Wang, X.; Wang, R.; Hao, Y.; Ren, F.; Wang, P.; Fang, B. Faecalibacterium prausnitzii Supplementation Prevents Intestinal Barrier Injury and Gut Microflora Dysbiosis Induced by Sleep Deprivation. *Nutrients* **2024**, *16*, 1100.
7. Kikuchi-Hayakawa, H.; Ishikawa, H.; Suda, K.; Gondo, Y.; Hirasawa, G.; Nakamura, H.; Takada, M.; Kawai, M.; Matsuda, K. Effects of *Lacticaseibacillus paracasei* Strain Shirota on Daytime Performance in Healthy Office Workers: A Double-Blind, Randomized, Crossover, Placebo-Controlled Trial. *Nutrients* **2023**, *15*, 5119.
8. Baba, Y.; Saito, Y.; Kadowaki, M.; Azuma, N.; Tsuge, D. Effect of Continuous Ingestion of Bifidobacteria and Inulin on Reducing Body Fat: A Randomized, Double-Blind, Placebo-Controlled, Parallel-Group Comparison Study. *Nutrients* **2023**, *15*, 5025.
9. Azuma, N.; Mawatari, T.; Saito, Y.; Tsukamoto, M.; Sampei, M.; Iwama, Y. Effect of Continuous Ingestion of Bifidobacteria and Dietary Fiber on Improvement in Cognitive Function: A Randomized, Double-Blind, Placebo-Controlled Trial. *Nutrients* **2023**, *15*, 4175.
10. Sheu, M.; Yeh, M.; Tsai, M.; Wang, C.; Chang, Y.; Wang, C.; Huang, H. Glucosinolates Extracts from *Brassica juncea* Ameliorate HFD-Induced Non-Alcoholic Steatohepatitis. *Nutrients* **2023**, *15*, 3497.
11. Chen, J.; Yang, N.; Peng, Y.; Zhou, H.; Li, Q. Association between Nonfood Pre- or Probiotic Use and Cognitive Function: Results from NHANES 2011–2014. *Nutrients* **2023**, *15*, 3408.
12. Anegkamol, W.; Kamkang, P.; Hunthai, S.; Kaewwongse, M.; Taweevisit, M.; Chuaypen, N.; Rattanachaisit, P.; Dissayabutra, T. The Usefulness of Resistant Maltodextrin and Chitosan Oligosaccharide in Management of Gut Leakage and Microbiota in Chronic Kidney Disease. *Nutrients* **2023**, *15*, 3363.
13. Pedrosa, L.; de Vos, P.; Fabi, J. From Structure to Function: How Prebiotic Diversity Shapes Gut Integrity and Immune Balance. *Nutrients* **2024**, *16*, 4286.
14. Guamán, L.; Carrera-Pacheco, S.; Zúñiga-Miranda, J.; Teran, E.; Erazo, C.; Barba-Ostria, C. The Impact of Bioactive Molecules from Probiotics on Child Health: A Comprehensive Review. *Nutrients* **2024**, *16*, 3706.
15. Di Costanzo, M.; Vella, A.; Infantino, C.; Morini, R.; Bruni, S.; Esposito, S.; Biasucci, G. Probiotics in Infancy and Childhood for Food Allergy Prevention and Treatment. *Nutrients* **2024**, *16*, 297.
